# Nutritional Stress in Head and Neck Cancer Originating Cell Lines: The Sensitivity of the NRF2-NQO1 Axis

**DOI:** 10.3390/cells8091001

**Published:** 2019-08-29

**Authors:** Lidija Milković, Marko Tomljanović, Ana Čipak Gašparović, Renata Novak Kujundžić, Dina Šimunić, Paško Konjevoda, Anamarija Mojzeš, Nikola Đaković, Neven Žarković, Koraljka Gall Trošelj

**Affiliations:** 1 Laboratory for Oxidative Stress, Division of Molecular Medicine, Ruđer Bošković Institute, 10000 Zagreb, Croatia; 2 Laboratory for Epigenomics, Division of Molecular Medicine, Ruđer Bošković Institute, 10000 Zagreb, Croatia; 3 University Hospital Centre Sisters of Charity, Institute for Clinical Medical Research and Education, 10000 Zagreb, Croatia; 4 Department of Clinical Oncology, School of Medicine, University of Zagreb, 10000 Zagreb, Croatia

**Keywords:** glucose deprivation, glutamine deprivation, viability, proliferation, ROS, NRF2-NQO1 axis, IMR-90, NQO1 transcript variants, rs1800566, TP53 mutation

## Abstract

Nutritional stress disturbs the cellular redox-status, which is characterized by the increased generation of reactive oxygen species (ROS). The NRF2-NQO1 axis represents a protective mechanism against ROS. Its strength is cell type-specific. FaDu, Cal 27 and Detroit 562 cells differ with respect to basal NQO1 activity. These cells were grown for 48 hours in nutritional conditions (NC): (a) Low glucose–NC2, (b) no glucose, no glutamine–NC3, (c) no glucose with glutamine–NC4. After determining the viability, proliferation and ROS generation, NC2 and NC3 were chosen for further exploration. These conditions were also applied to IMR-90 fibroblasts. The transcripts/transcript variants of *NRF*2 and *NQO*1 were quantified and transcript variants were characterized. The proteins (NRF2, NQO1 and TP53) were analyzed by a western blot in both cellular fractions. Under NC2, the NRF2-NQO1 axis did not appear activated in the cancer cell lines. Under NC3, the NRF2-NQO1axis appeared slightly activated in Detroit 562. There are opposite trends with respect to TP53 nuclear signal when comparing Cal 27 and Detroit 562 to FaDu, under NC2 and NC3. The strong activation of the NRF2-NQO1 axis in IMR-90 resulted in an increased expression of catalytically deficient NQO1, due to NQO1*2/*2 polymorphism (rs1800566). The presented results call for a comprehensive exploration of the stress response in complex biological systems.

## 1. Introduction

To date, the roles of glucose and glutamine in the biology of transformed cells both in vitro and in vivo, have been evaluated in various cellular systems, most often as separate entities. It is well-established that cancer cells need glucose as a source of carbon. They also need glutamine. Not only as an alternative substrate for the Krebs cycle and ATP production, but also as a source of carbon and nitrogen, glutamine is needed for various biosynthetic reactions and glutathione production to support antioxidant defense [1].Under normoglycemic conditions, the generation of the fundamental metabolite nicotinamide adenine dinucleotide phosphate (NADPH) is secured via the glucose catabolism pathway—the pentose phosphate pathway (PPP). Glucose starvation results in decreased ATP production and could induce oxidative stress by downregulating NADPH production by PPP. Under these conditions, metabolic reprogramming and redox regulation are closely related to the activation of 5′ AMP-activated protein kinase (AMPK) pathway. It is a protective mechanism aimed at prolonging cell survival by preventing excessive NADPH consumption in fatty acid synthesis and increasing NADPH generation in the process of fatty acid oxidation [2]. When the protective capacity of the AMPK pathway is exceeded, the regeneration rate of glutathione decreases, and there is an increase in ROS, an indicator of the disbalanced cellular redox status. It is well-known that ROS are involved in the nutrient deprivation-induced Warburg effect [3]. Glutamine has also been shown as a source of NADPH. In pancreatic cancer cells with a K-RAS-regulated metabolic pathway, the glutamine-derived malate can be converted to pyruvate by malic enzyme. This reaction is associated with NADPH generation [4]. It has also been shown recently that a lack of glutamine may promote rapid and transient activation of AMPK [5].

During glucose deprivation, activated AMPK phosphorylates the wild type (WT) TP53 at serine 15, leading to G1/S cell cycle arrest and cellular senescence [6]. WT TP53 has an important role in cellular metabolism. It inhibits the monomeric form of the enzyme glucose-6-phosphate dehydrogenase (G6PD), which is present in the cytoplasm. This event results in one more instance of NADPH depletion. The effect seems to be characteristic of WT TP53, but not its mutant forms. It was proposed as a main function of cytoplasmic WT TP53 in resting cells [7].

When deprived of oxygen and glucose, the cells activate the AMPK by NAD(P)H:quinone oxidoreductase 1 (NQO1) [8]. This enzyme was purified and characterized for the first time in 1988 [9]. It was originally considered only as a flavin adenine dinucleotide (FAD)—dependent, two-electron reductase. There are numerous proofs of its effectiveness, associated with reducing quinones to hydroquinones through a two-electron transfer. The catalytically active form of the enzyme is a homodimeric protein. It has two identical active sites located at the interface between monomers and with one FAD bound per monomer. Each of these two sites is shared by both reduced pyridine nucleotide cofactors, NADH and NADPH [10]. The model of the NQO1 mode of action (ping-pong-bi-bi kinetic mechanism), proposed in 1974 [11], is still considered valid. The catalytic cycle is initiated by the binding of reduced pyridine nucleotide in the active site, followed by a hydride transfer to FAD. It leads to a conformational change expelling the oxidized pyridine nucleotide, nicotinamide adenine dinucleotide (NAD+), and creating an environment for quinone binding. The generation of NAD+ makes a strong, functional, yet indirect link between NQO1 and two very important cellular enzymes relevant for metabolism and metabolic reprogramming in cancer. These are NAD+ dependent sirtuin 1 (SIRT1) and PARP-1, a major NAD+-consuming enzyme [12].

The enzymatic activity of NQO1 can be detected in the cytosol and in the nucleus [13]. It has an important role in eliminating free radicals [14] which increase during nutritional stress. According to the most recent data, NQO1 is a central unit of the redox-dependent switch. It depends on NQO1 conformational change, in which NADH has strong protective role against tryptic digestion and loss of the C-terminal NQO1 domain. To a lesser extent, a protective role was also obtained with NADPH [15].

Altered pyridine nucleotide ratios could induce a switch in protein conformation. This results in binding of NQO1 to a different set of proteins and RNA under oxidative conditions [16]. Thus, NQO1 action influences the activity of other proteins indirectly, through generating NAD+ (SIRT1, PARP-1) [17] and through direct binding (hypoxia-inducible factor, alpha subunit, HIF1-α, TP53) [18,19].

NQO1 stabilizes both wild-type (WT) [18] and mutant-types (MT) TP53 protein [20] by protecting them from the ubiquitin-independent 20S proteasomal degradation. This stabilizing effect is most prominent under oxidative stress. However, the presence of the single nucleotide variation (SNV) rs1800566 that occurs in NQO1 exon 6, strongly decreases the enzymatic activity of NQO1 and abolishes TP53 stability mediated by NQO1 [21,22]. This polymorphism, also known as NQO1*2 (heterozygote)/NQO1*2/*2 (homozygote), was shown to be an important factor in a poor clinical response to quinone (mitomycin C, β-lapachone)-based chemotherapy. This is due to a lack of drug bioactivation [23,24].

TP53, which is traditionally considered a tumor suppressor, is currently an emerging research topic relating to nutritional stress [25]. Its connection to NQO1 may be a critical factor for cellular adaptive stress response, especially during nutrient deprivation. The most recent data have shown that the withdrawal of glutamine activates TP53 [26]. In a glutamine deprived cell, TP53 binds to the promoter of the solute-like carrier family 7, member 3 (SLC7A3). It promotes cancer cell adaptation to glutamine deprivation by upregulating SLC7A3to increase arginine uptake [27].

The state of oxidative stress is of utmost importance for activating *NQO*1 transcription, which is mediated by NFE2L2 (Nuclear Factor, Erythroid 2 Like 2: NRF2). When there is an excess of ROS, NRF2 dissociates from its cytoplasmic partner Kelch-Like ECH-Associated Protein 1 (KEAP-1). It enters the nucleus and binds to the *cis*-acting elements in an array of NRF2 target genes called antioxidant response elements (AREs) [28]. These are present in the *NQO*1 promoter [29]. Consequentially, this event leads to an increased transcriptional activity of the *NQO*1 gene. This phenomenon has been shown in various models as a part of a strong antioxidative cellular response.

**Figure 1:**
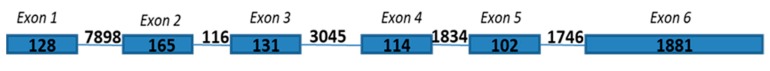
Structure of the *NQO*1 gene, NG_011504.2.

One very interesting molecular-genetic aspect of *NQO*1 mRNA is associated with the deposit of four NQO1 transcript variants (TVs) in the GeneBank. The gene itself contains six exons (Figure 1). All of them are part of the longest transcript (TV1; NM_000903.3, N = 2521 nt). Another three transcripts are characterized, as follows: TV2: NM_001025433.2; exon 5 excluded (N = 2419 nt); TV3: NM_001025434.2, exon 4 excluded (N = 2407 nt); TV4: NM_001286137.2, exons 4 and 5 excluded (N = 2305 nt).

In 1995, Gasdaska et al. described the *NQO*1 transcript lacking exon 4 (TV3) in cancer cell lines SW 480 and HT-29. The existence of the corresponding protein was not confirmed [30]. Seven years later, it was proposed that the polymorphism present at the end of exon 4, rs1131341 (Arg137Trp, also known as NQO1*3*), has a strong influence on NQO1 splicing. As a consequence, the ratio TV1/TV3 (shown by end-point PCR to be around 9.0 in NQO1*1/*1, NQO1*2/*1, NQO1*2/2*), significantly decreases (TV1/TV3 = 2) [31]. The ratio of TVs may vary depending on stressful conditions [30]. This was shown only once, in the mononuclear cells obtained from patients before and at various times following treatment with mitomycin [32]. According to SwissProt, there is only one experimentally verified NQO1 protein variant which is coded by NQO1 TV1. It consists of 274 amino acids (30.868 kDa).

As recently shown in a yeast model, introns negatively regulate growth in a rich medium. They are clearly required for maintaining cellular viability during the deprivation of nutrients (dextrose and phosphates) [33]. In 2007, Pleiss et al. showed that two different stress-inducers (ethanol exposure and amino acids deprivation) induce unique splicing profiles. This suggests that in yeast at least two independent pathways connect the spliceosome with the cellular environment [34].

Alternative splicing was shown to take place during nutrient depletion in an organoid model system derived from murine intestinal epithelial cells. This included exon skipping events and events involving full intron retention (IR-S; intron retention simple) and complex intron retention (IR-C; intron retention complex) [35].

Based on these facts, we wanted to explore selected cellular parameters (cellular viability and proliferation rate, ROS generation) and molecular events included in the axis NRF2-NQO1/TP53, under two different forms of nutritional stress. The transcripts (quantitatively - NRF2, NQO1 and qualitatively - NQO1 splice variants) and proteins (NRF2, NQO1, TP53) in cytoplasmic and nuclear cellular fractions were validated. Three cell lines originating from the head and neck squamous cell carcinomas (HNSSC) were used: FaDu; Cal 27; and Detroit 562. These cells significantly differ with respect to basal NQO1 activity (FaDu > Detroit 562 > Cal 27) [36]. IMR-90 fibroblasts, which are considered as NQO1 non-expressing cell lines [37], were used as representative of an untransformed cell line.

## 2. Materials and Methods

### 2.1. Cell Lines and Cell Culture Conditions

The cells originating from metastatic pharyngeal cancer (pleural effusion-Detroit 562) and human fetal lung fibroblasts (IMR-90) were purchased from Sigma-Aldrich (St. Louis, MO, USA). The human tongue squamous carcinoma cells (Cal 27) and human hypopharyngeal squamous carcinoma cells (FaDu) were purchased from the American Type Culture Collection (ATCC, LGC Standards GmbH, Wesel, Germany). The cells were cultured in T75 cell culture flasks (Sarstedt AG&Co.KG, Nümbrecht, Germany), in Dulbecco’s Modified Eagle’s Medium (DMEM, D5796; Sigma-Aldrich, St. Louis, MO, USA), supplemented with a 10% fetal bovine serum (FBS, Sigma-Aldrich, St. Louis, MO, USA), without antibiotics, at 37 °C in a humidified atmosphere and in the presence of 5% CO_2_. Prior to the experiments, the cells were harvested with 0.25% (*w*/*v*) Trypsin-0.53 mM EDTA (Ethylenediaminetetraacetic acid) solution and counted with the trypan blue exclusion assay in Bürker-Türk hemocytometer (Brand, Wertheim, Germany). For the experiments performed, the cells were cultured in DMEM, under the following four nutritional conditions (NCs) with respect to glucose and glutamine: NC1-high glucose (4.5 g/L) with l-glutamine (0.584 g/L) (D5796; Sigma-Aldrich, St. Louis, MO, USA); NC2-low glucose (1 g/L) with l-glutamine (0.584 g/L) (D6046, Sigma-Aldrich, St. Louis, MO, USA); NC3-no glucose and no glutamine (A14430, Gibco, Life Technologies Corporation, Grand Island, NY, USA); NC4-no glucose (A14430), but with 0.584 g/L of l-glutamine (Sigma Aldrich, St. Louis, MO, USA).

### 2.2. Cell Viability Assay

Cellular viability was measured using EZ4U assay (Biomedica, Vienna, Austria), which assesses cellular viability through reducing tetrazolium salts to colored formazan derivatives in the mitochondria of living cells. The cells were seeded in 96-well plates (TPP, Trasadingen, Switzerland) at a density of 1×10^4^ cells per well and cultivated in 200 μL of previously described media formulations (NC1-NC4), supplemented with 10% FBS. After a cultivation period of 48 hours, 20 μL of the dye substrate (tetrazolium salts) was added to each well. After a 2 h incubation, formazan derivatives were quantified by measuring the absorbance using the microplate reader Multiskan EX (Thermo Electron Corporation, Shanghai, China) at 450 nm, with 620 nm as a reference wavelength. Cellular viability under tested conditions was expressed as a percentage of the viability of the control cells (cells grown in a high glucose + glutamine, NC1, medium).

### 2.3. Cell Proliferation Assay

The rate of cellular proliferation was estimated through incorporating pyrimidine analogue BrdU (5-bromo-2′-deoxyuridine), in place of thymidine, into the DNA of proliferating cells, using the Cell Proliferation ELISA, BrdU (colorimetric) Kit (Roche Applied Science, Mannheim, Germany). The antibody conjugated anti-BrdU-peroxidase binds incorporated BrdU. The complex BrdU/anti-BrdU-peroxidase was detected by the reaction between peroxidase conjugated to the BrdU antibody and the substrate (3,3',5,5'-tetramethylbenzidine). After reaching a satisfactory color intensity (after incubating between 5 and 30 min), the reaction was stopped with 1 M H_2_SO_4_ solution.

The cells were seeded in 96-well plates (TPP, Trasadingen, Switzerland) at a density of 1×10^4^ cells per well and were maintained in 200 μL of previously described media formulations (NC1-NC4), supplemented by 10% FBS. After 48 h of incubation, the assay was performed according to the manufacturer's instructions. The reaction product (3,3',5,5'-tetramethyl-benzidine diimine) was quantified by measuring absorbance using a microplate reader Multiskan EX (Thermo Electron Corporation, Shanghai, China) set at 450 nm (reference wavelength: 620 nm). Cell proliferation was expressed as a percentage of the cells grown under condition NC1 (high glucose + l-glutamine medium).

### 2.4. ROS Measurement

The intracellular levels of reactive oxygen species (ROS) were detected by DCFH-DA (2ʹ,7ʹ-Dichlorofluorescin Diacetate; Sigma-Aldrich, St. Louis, MO, USA). The cells were seeded in white 96-well plates (Thermo Fisher Scientific, Nunc A/S, Roskilde, Denmark) at a density of 1×10^4^ cells per well and maintained in 200 μL of the previously described media formulations (NC1-NC4), supplemented by 10% FBS. After growing for 48 hours, the cells were incubated with 20 μL of 100 μM DCFH-DA, which was added to the culture media. After 45 min of incubation, the medium containing DCFH-DA was replaced with 200 μL of fresh medium. The fluorescence intensity was measured immediately (zero point) and after one hour, on a plate reader Infinite 200 PRO (Tecan Group Ltd., Männedorf, Switzerland). The excitation/emission wavelengths for DCFH-DA were set at 500/529 nm. The values of the emitted fluorescence were expressed as arbitrary units, which represent the difference between the two points of measurement (one hour and zero point). Additionally, the values were corrected with respect to the cell numbers, which varied in relation to the treatment applied.

### 2.5. Nucleic Acids Extraction

The cells were cultured for 48 h at a density of 1×10^6^ in T25 flasks (Sarstedt AG&Co.KG, Nümbrecht, Germany), in 5 mL of the previously described media formulations (NC1-NC3), supplemented by 10% FBS.

The total RNA was extracted from the cells cultivated and treated in 25 cm^2^ flasks (Sarstedt AG&Co.KG, Nümbrecht, Germany). The medium was removed and extraction was performed by TRIzol (Invitrogen, Carlsbad, CA, USA), according to the manufacturer’s instructions. The integrity of isolated RNA was determined by electrophoresis, on 1% agarose gel stained with ethidium bromide (EtdBr) (Sigma-Aldrich, St. Louis, MO, USA). As there were no issues relating to the integrity of the extracted RNA, all samples were further purified with gDNA Removal Kit (Jena Bioscience, Jena, Germany), according to the manufacturer’s instructions. The concentration and purity of extracted RNA was determined spectrophotometrically (BioSpec-nano, Shimadzu Biotech, Japan) by measuring the absorbance at the following wavelengths: 230, 260 and 280 nm. The samples were stored at −80 °C.

The genomic DNA was extracted by phenol-chloroform extraction, after an overnight incubation with Proteinase K (QIagen, Holden, Germany), as previously described [38]. After successful precipitation, the samples of extracted DNA were re-suspended in TE buffer (10 mM Tris, 1 mM EDTA, pH 7.4). The concentration and quality of the extracted DNA was determined spectrophotometrically and electrophoretically, in 1% gel agarose stained with EtdBr. The samples were stored at +4 °C.

### 2.6. Construction of Primers

For all primers used in this research, with the exception of GAPDH1/GAPDH2 which are commonly used, the modeling through combining the programs Primer-BLAST and Primer3Plus were performed. Table 1 shows the primer sequences, their exact position on the RefSeq and the expected amplicon sizes.

The composition of the nucleotides of the primers used allowed the authors to perform the polymerase chain reaction under almost identical conditions. The primers for gDNA were selected to anneal to the template at 58 °C, while the primers for cDNA annealed to the template at 59 °C.

The three primers for GAPDH were constructed in a way which allowed combining the primer GAPDH2 with primers GAPDH1—for determining cDNA quality and GAPDH3—for a rigorous check of the potential gDNA contamination. These strict precautionary measures were undertaken because the TaqMan probe used for quantifying NRF2 may bind to the gDNA, at least according a statement provided.

The selection of primers which would allow for the amplification of all four NQO1 TVs in one reaction was based on the primary structure of the NQO1 TV1 mRNA (Figure 1). The primers were complementary to the stretch of nucleotides positioned in the 3′ region of the exon 2 (NQO1F) and 5′ region of the exon 6 (NQO1R), respectively. All primers which allowed for examining the exon/intron boundaries sequences were used only on genomic DNA.

### 2.7. Reverse Transcription, RT – PCR, and PCR

The reverse transcription was performed with a High-Capacity cDNA Reverse Transcription Kit (Thermo Fisher Scientific, Waltham, MA, USA), with anchored Oligo(dT)_23_ primers (Sigma-Aldrich, St. Louis, MO, USA) and 1 µg of total RNA in a 20 μL volume, according to the manufacturer’s instructions. The reaction conditions were: 25 °C/10 min; 37 °C/120 min; 85 °C/5 min; 4 °C/indefinite. After finalization of the reverse transcription, 80 μL of sterile, deionized water was added to the tubes to achieve a total volume of 100 μL of cDNA, which was used for subsequent reactions.

The efficacy of reverse transcription was assessed with the end-point polymerase chain reaction (PCR) using the primer pair GAPDH 1/GAPDH 2 and 1 μL of diluted cDNA. This template volume was used as a standard in all end-point PCR reactions. For discovering the potentially present traces of contaminating DNA, the primer pair GAPDH2/GAPDH3 was used, as the sequence of the GAPDH3 primer is complementary to the nucleotides in intron 5. The polymerase chain reaction was carried out in GeneAmp PCR System 2400 (Applied Biosystems, Foster City, CA, USA). The reaction mixture (12.5 μL) contained AmpliTaq 360 Gold Master Mix and GC Enhancer (Thermo Fisher Scientific, Waltham, MA, USA), home-made nuclease free-water and primers (final concentration: 400 nM).

The genomic DNA was amplified with the same sets of chemicals and in the same volume, with 200 ng of gDNA. The reaction conditions were: Predenaturation 95 °C/5 min, followed by 35 cycles: 9 °C/30 s; 58 °C and 59 °C for gDNA and cDNA, respectively/30 s, 72 °C/30 s. The final elongation was at 72 °C, for 7 min.

### 2.8. Densitometry, Purification of PCR Products from Agarose Gel and Sequencing

The PCR products were separated by electrophoresis in 2% agarose gel and photographed for future densitometric analysis, which was performed with ImageJ [39]. Bands of interest were cut out of the gel and purified using a GenElute Gel Extraction Kit (Sigma-Aldrich, St. Louis, MO, USA), according to the manufacturer’s instructions. After being eluted from the column, 10 µL of eluate was loaded in 2% agarose gel in order to determine the purity and amount of eluate that was going to be used in the sequencing reaction. Only the amplicon corresponding to TV4 needed to be purified, re-amplified one more time with NQO1F/NQO1R and purified again. A sufficient amount of the template was obtained for the sequencing reaction only after this additional procedure.

The sequencing reaction contained 12 µL mixture of the purified PCR product (approximately 50 ng per 100 bps), nuclease-free water and 1 µL of the forward and reverse primer used in PCR, respectively (primer concentration 3.2 pmol/µL), for a total volume of 13 µL. The samples were sequenced at the DNA Sequencing Core Facility of the Rudjer Boskovic Institute.

### 2.9. Real-Time RT-PCR and Rationale for Specific TaqMan’s Probe Selection

For quantification of NQO1 and NRF2, TaqMan chemistry was used. The reactions were performed in a 7300 Real-Time PCR System (Applied Biosystems, Foster City, CA, USA). We used always 1.5 μL of cDNA template, 10 μL of TaqMan Fast Advanced Master Mix (Thermo Fisher Scientific, Waltham, MA, USA), 1 μL of the probe and 7.5 μL of sterile, deionized water. The reactions were performed in triplicate for each template and for each probe and in at least three biological replicates, which we tried to associate with three consecutive passages. Microamp 96-well rxn plates (Thermo Fisher Scientific, Waltham, MA, USA) were used, and amplification was performed under the following conditions: Incubation 50 °C/2 min + 95 °C/10 min, followed by 40 cycles; 95 °C/15 s, 60 °C/60 s. The following probes were used for house-keepers: GAPDH—Hs99999905_m1 (as GAPDH was used in the end-point PCR reactions); TBP—Hs00920495_m1 (as the TBP was used in the WBs); HPRT1—Hs02800695_m1 (as it was shown to be relatively stable in some published studies) [40].

For relative quantification, delta-delta Ct 2–∆∆Ct developed by Applied Biosystems was used, which presumes identical amplification efficiencies of the target and reference gene and the Pfaffl method [41], which served as a correctional factor in subsequent calculations.

### 2.10. Protein Extraction and Western Blot Analyses

The cells were cultured for 48 hours at a density of 1×10^6^ in T25 flasks (Sarstedt AG&Co.KG, Nümbrecht, Germany), in 5 mL of the previously described media formulations (NC1-NC3), supplemented by 10% FBS. The proteins were extracted with NE-PER nuclear and cytoplasmic extraction reagents (Thermo Scientific -Pierce Biotechnology, Rockford, IL, USA), supplemented by the protease inhibitor (Complete Mini Protease Inhibitor Cocktail Tablets; Roche Applied Science, Mannheim, Germany). The protein amount was estimated using the Bradford method [42]. The absorbance was measured at 595 nm using the microplate reader Multiskan EX (Thermo Electron Corporation, Shanghai, China). The protein samples were mixed with Laemmli buffer and boiled for 5 min at 95 °C. The equal amounts of protein (10 μg) were loaded on the gel (9% resolving and 5% stacking), separated electrophoretically and transferred to nitrocellulose membranes (Roti®-NC, Carl Roth, Karlsruhe, Germany). The transfer efficacy was evaluated by staining the membranes with Ponceau S solution (Sigma Aldrich, St. Louis, MO, USA). The stained membranes were scanned. After incubating the membranes with 5% nonfat milk (Cell Signaling Technology, Danvers, MA, USA) in Tris-buffered saline (TBS; 50 mM Tris-Cl, 150 mM NaCl, pH 7.6) containing 0.1% Tween-20 for 1 h, the membranes were probed overnight with the following primary antibodies: (all rabbit monoclonal, Cell Signaling Technology, Danvers, MA, USA): anti-NQO1 (1:1000; CST: #62262); anti-NRF2 (1:1000; CST:#12721); anti-TP53 (1:1000; CST:#2527); anti-TBP (1:1000; CST:#44059); anti-β-actin (1:1000, CST:#8457). The last two antibodies were used as the loading controls for nuclear and cytoplasmic fractions, respectively. The expected molecular weights of the detected proteins were: NQO1—29 kDa; NRF2—97–100 kDa; p53—53 kDa; TBP 35–45 kDa; β-actin 45 kDa. After three washings of the membranes with TBST (0.1% Tween 20 in 1× TBS), the immunoreactive bands were detected with an HRP- linked anti-rabbit IgG secondary antibody (1:2000; CST: #7074). The immunological complexes were visualized using SuperSignal^TM^ West Pico PLUS Chemiluminescent Substrate (Thermo Scientific, Rockford, IL, USA) and Alliance 4.7 (UVITEC, Cambridge, UK). The protein expression levels were quantified using ImageJ and/or Image Studio Lite (LI-COR, Lincoln, NE, USA) analysis software. The relative change of signals obtained was calculated after normalization according to the loading controls and Ponceau S signals.

### 2.11. Statistical Analyses

Each experiment related to cellular biology (viability, proliferation, ROS generation) was performed in technical triplicates or quadriplicates and repeated three times (as specified in the Figure legend). The data obtained was analyzed with 1-way ANOVA and Tukey post-hoc test, as indicated in the figure legends. The same principle was applied for producing and analyzing the data obtained with molecular biology methods in biological triplicates. For both analyses and visualization, GraphPad 6.0 was used. The statistical significance of the differences obtained for all data analyzed was considered significant at *p* < 0.05.

## 3. Results

Nutritional conditions were first profiled using the cancer cell lines originating from HNSCC: FaDu; Cal 27; Detroit 562. In this study, highly proliferating cells were used for exploringbasic cellular parameters before including a very slowly proliferating cell line—IMR-90 fibroblasts.

### 3.1. Viability, Proliferation and Generation of ROS

**Figure 2:**
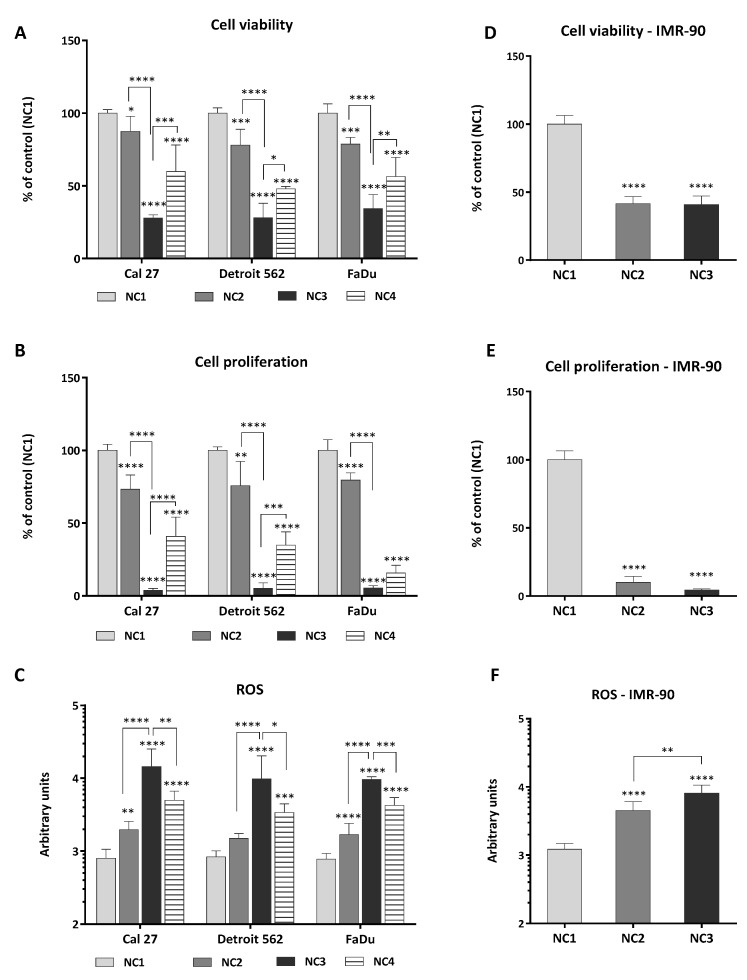
The viability, proliferation and generation of ROS in cancer cell lines (**A**–**C**) and IMR-90 (**D**–**F**) after exposure to NC1-NC4 and NC1-NC3, respectively, for 48 hours. One-way ANOVA with Tukey post-hoc test was used to test the differences with regard to nutrient conditions. The values are shown as the mean ± 95% CI. N = 3. * *p* < 0.05; ** *p* < 0.01; *** *p* < 0.001; **** *p* < 0.0001.

The choice of experimental conditions needed for nutritional stress induction, in relation to the concentration of glucose and glutamine in the medium, was combined with measuring cellular viability (Figure 2A), cellular proliferation (Figure 2B), and the amount of ROS generated at hour 48 (Figure 2C). Initially, the four nutritional conditions (NC1-NC4) were established, as described in the Material and Methods section, to which only the cancer cells were exposed. Then, based on the data obtained, this research was extended to IMR-90, through applying nutritional conditions NC2 and NC3. NC1 should be considered the control condition.

As presented in Figure 2A, the viability of the cancer cell lines, regardless of their genetic background, was similar under the given conditions. Predictably, the most intensive decrease in cellular viability (up to 70%) was recorded for all three cancer cell lines in the medium without glucose and glutamine (NC3), when compared to both NC1 and NC2 (*p* < 0.0001).

The presence of glutamine in a medium without glucose (NC4) was beneficial for the viability of all cancer cell lines (Figure 2A). It was also beneficial for the cellular proliferative capacity (Figure 2B) of Detroit 562 (*p* = 0.0007) and Cal 27 (*p* < 0.0001), but not FaDu. The strongest ROS generation was associated with condition NC3. The presence of glutamine in a medium without glucose (NC4) led to a decreased ROS generation in all three cancer cell lines (Figure 2C). Under NC2, the generation of ROS in Cal 27 and FaDu was stronger (*p* = 0.0055 and *p* < 0.0001, respectively) than in Detroit 562 (Figure 2C).

When reviewed, these data indicated that the cancer cell lines showed some interesting and unique features. Under NC2, the generation of ROS was not significantly increased only in Detroit 562. Under NC4, when there is a lack of glucose, FaDu was far less sensitive to the rescuing effect of glutamine on proliferative capacity.

These data are also very indicative regarding the degree of cellular sensitivity to glutamine deprivation, showing that Cal 27 and Detroit 562 were more dependent on glutamine than was FaDu.

Knowing that non-transformed cells are highly dependent on glucose, and relying on the data obtained with the cancer cell lines (Figure 2A–C), we chose to continue the experiments using the mildest (NC2) and the most robust condition (NC3), now including the IMR-90 fibroblasts.

They were considered a good control system, to compare with the cancer cell lines. The Figure 2D–F represent IMR-90 response to NC2 and NC3. The viability of IMR-90 was unique in the extreme sensitivity of this cell line to the mild glucose deprivation, during 48 hours (NC2; *p* < 0.0001) (Figure 2D). The viability and proliferative capacity after 48 hours in NC2 (Figure 2D,E) seems to be a maximal effect of a critical nutrient deprivation (glucose) because 48 h of cultivation in the medium without glucose and glutamine (NC3) did not influence these cellular parameters further. However, the generation of ROS did differ between NC2 and NC3 (*p* = 0.0019) (Figure 2F), although not as strong as in the cancer cell lines (*p* < 0.0001) (Figure 2C). The lack of a significant change of IMR-90 viability after 48 hours of exposure to mild and extreme starvation, is in clear contrast with the cancer cell lines and needs to be further explored.

### 3.2. Quantification of NQO1 and NRF2 in a Real-Time

According to the majority of literature data, the NRF2-NQO1 axis should be highly active under conditions which induce the generation of ROS. As a first step forward, the transcriptional activation of *NRF*2 and *NQO*1 in real-time was explored. Three different house-keeping genes were used, as we were aware that under the experimental conditions we chose to explore, we might not be able to make an accurate quantification of our targets. Under the given conditions, none of the three housekeepers (*GAPDH*, *TBP*, *HPRT*1) used in this research were universally stable in all four cell lines. The changes in their transcription rates were clearly both condition specific and cell-type specific, as already presented [40]. Finally, under the given conditions, a quantification was made by combining these three housekeepers in a particular cell line.

**Figure 3:**
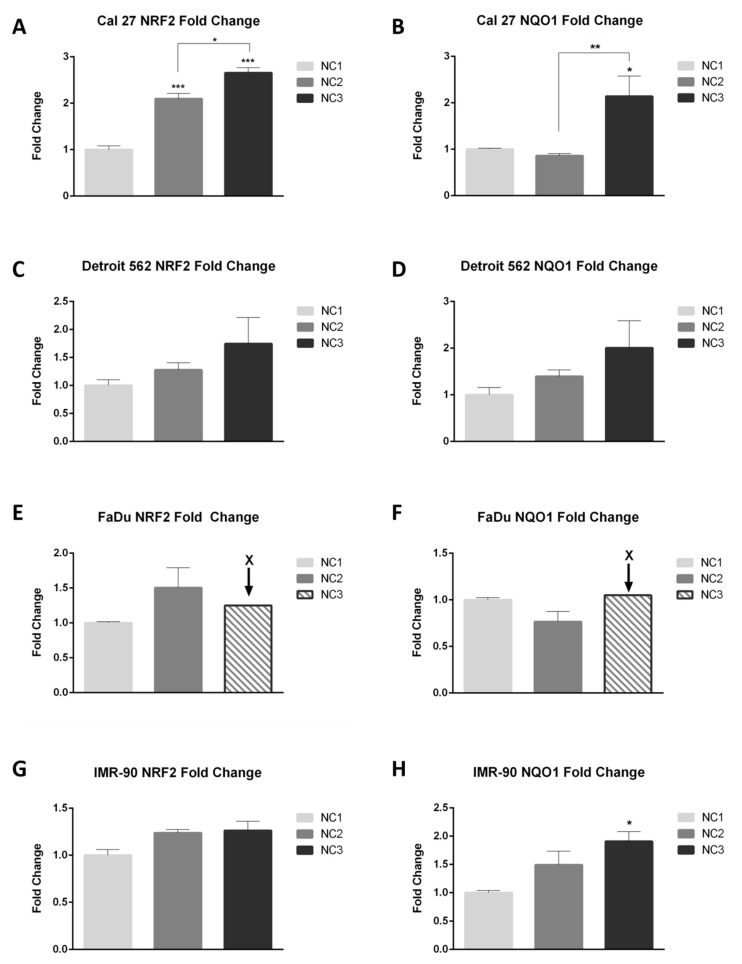
The quantification of target transcripts, *NRF*2 and *NQO*1, in real time (RT-qPCR). One-way ANOVA with Tukey post hoc test was used to test the differences with respect to the quantity of *NRF*2 and *NQO*1 mRNA, under different nutritional conditions. The values are shown as the mean ± SD. N = 3. * *p* < 0.05; ** *p* < 0.01; *** *p* < 0.001; **** *p* < 0.0001. * The Fold Change for NRF2 and NQO1 in FaDu under NC3 was estimated according to the Ct values.

The general picture shows that in all cell lines, the transcriptional activity of *NRF*2 increases under NC2 and NC3 (Figure 3A,C,E,G). The quantification for FaDu, under NC3, was estimated based on the Ct values for NRF2 and NQO1 under NC1 and NC2, in respect to NC3 (Figure 3E,F). However, under NC2, the increasing trends of *NQO*1 were not present in Cal 27 and FaDu. The statistically significant differences in transcription rates were reached only in Cal 27, for both genes: *NRF*2: NC1 versus NC2; *p* = 0.0008; NC2 versus NC3; *p* = 0.0102: *NQO*1: NC1 versus NC3; *p* = 0.0153; NC2 vs. NC3; *p* = 0.0072 and IMR-90, but for *NQO*1 only: NC1 versus NC3; *p* = 0.0276. Notwithstanding the mathematical calculation, it is visible that only in Cal 27 and already at NC2 does the Fold Change of *NRF*2 reach a value above 2 (Figure 3A). Thus, it is quite obvious that the transcriptional level of *NRF*2, with the exception of Cal 27, does not exhibit as strong changes as expected, especially not with respect to the increased generation of ROS (all cancer cell lines: NC2 versus NC3 *p* < 0.0001; Figure 2C). An increase of *NQO*1 mRNA was recorded under NC3 in Cal 27, Detroit 562 and IMR-90. Due to significant variations in the transcriptional level of house-keepers in FaDu under NC3, the target transcripts were not accurately quantified, under that condition and only in that cell line.

### 3.3. Three Splice Variants of NQO1 are Present in All Four Cell Lines, Notwithstanding the Experimental Conditions Applied

**Figure 4:**
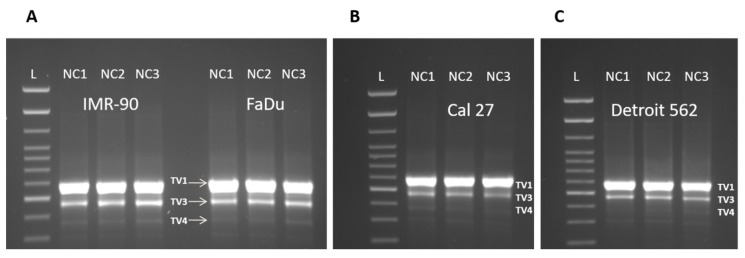
The presence of three NQO1 splice variants in all four cell lines, under three nutritional conditions (35 PCR cycles). L: 100 bp DNA ladder (Invitrogen). **A**. IMR-90 and FaDu; **B**. Cal 27; **C**. Detroit 562. TV1: 685 bps, TV3: 571 bps and TV4: 469 bps.

**Figure 5:**
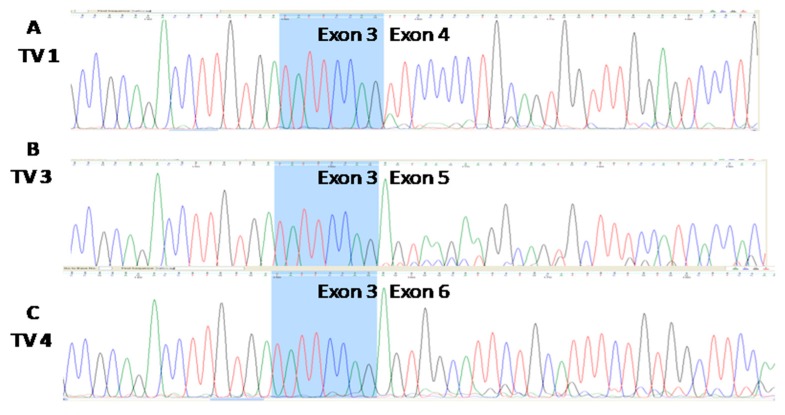
The sequence analyses of amplicons obtained from the Cal 27 NQO1-cDNA amplified with the primer pair NQO1F/NQO1R (Table 1), presented in Figure 4, confirm the presence of transcript variants TV1 (**A**), TV3 (**B**), and TV4 (**C**). Eight nucleotides in the terminal part of the 3’ exon 3 are common to all three amplicons and are shown in blue. The sequences of TVs were identical in all cell lines.

**Figure 6:**
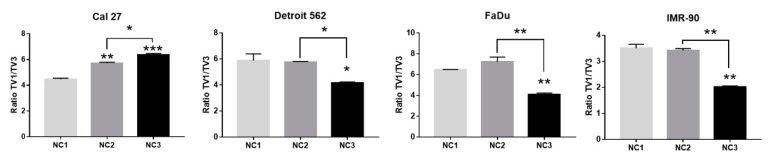
The dependence of the TV1/TV3 ratio on NCs applied. One-way ANOVA with Tukey post-hoc test. The ascending trends were recorded in Cal 27 as contrasted with the other three cell lines. The values are shown as the mean ± SD. N = 3. * *p* < 0.05; ** *p* < 0.01; *** *p* < 0.001; **** *p* < 0.0001.

In all four cell lines, three reproducible bands occurred in the end-point PCR (35 cycles), regardless of the cellular background and/or the type of nutritional stress (Figure 4A–C). Based on their size, we were certain of the existence of TV1 and TV4. The nature of the amplicon which was in the middle position (as the length of TV2 and TV3 differ for only 13 nucleotides, Figure 1) was revealed only after analyzing the sequencing data (Figure 5). Under all experimental conditions and in all cell lines, TV1 gave the most prominent signal and TV3 was visible. However, in Detroit 562, TV4 varied and was almost undetectable. There was an additional 400 bps long amplicon present in all cell lines under all conditions, which was not characterized further. After a densitometric analysis of the signals after 28 cycles (when the signal was less saturated) using the ImageJ, the changes in the ratio TV1/TV3 were recorded (Figure 6).

The signal obtained from TV4 was permanently low in all cell lines, under all NCs applied, and was excluded from the analyses. The intensity of the densitometric signal originating from TV1 and TV3 was expressed as their ratio (TV1/TV3) obtained after 28 PCR cycles (Supplementary Figure S1). The opposite trends in Cal 27 versus three other cell lines were observed. In Cal 27, the TV1/TV3 ratio increased from 4.47 (NC1) to 5.7 (NC2) and 6.36 (NC3) (Figure 6). The differences were statistically significant: NC1 versus NC2: *p* = 0.0020; NC1 versus NC3: *p* = 0.0006; NC2 versus NC3: *p* = 0.0123. In IMR-90, Detroit 562 and FaDu, the TV1/TV3 ratio was significantly decreased under NC3 (NC1 versus NC3; IMR-90: *p* = 0.0014; Detroit 562: 0.0213; FaDu: *p* = 0.0069). There were also significant differences between the ratio of TVs which was observed between NC2 and NC3 (IMR-90: *p* = 0.0017; Detroit 562: *p* = 0.0261; FaDu: 0.0031).

### 3.4. Detection of SNVs in Intron/Exon Boundaries

#### 3.4.1. rs 689460, G+C, Is Present in IMR-90 and Does Not Influence the NQO1 Splicing

**Figure 7:**
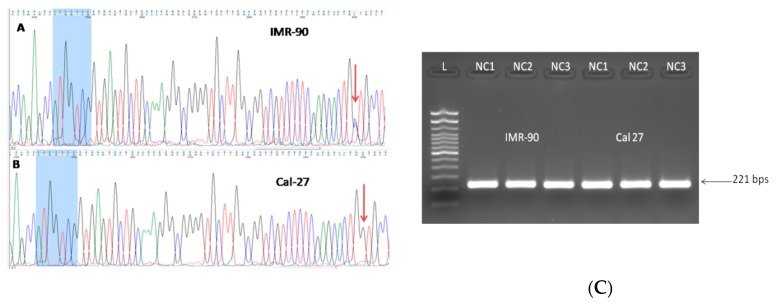
**A**. Single nucleotide variation C+G wass present in IMR-90, in NQO1 intron 1. The terminal part of the 3' exon 1 is shown in blue. The distance between the polymorphic locus, revealed to be rs 689,460 according to NCBI, and 3' of exon 1, is only 50 nts. **B**. All three cancer cell lines were homozygos, rs 689,460 G+G, as shown here, for Cal 27. **C**. Single nucleotide variant C+G in IMR-90 did not influence the NQO1 splicing. The amplicons obtained in IMR-90 under given conditions did not differ from those obtained in homozygos (G+G) cancer cell lines (only Cal 27 is shown). L: 100 bp DNA ladder. Lines 1–3 and lines 4–6: IMR-90 and Cal 27 under NC1, NC2 and NC3, respectively.

Based on the one paper showing that the polymorphism present at the very end of *NQO*1 exon 4 (rs1131341) favors occurrence of the transcript lacking exon 4 [31], and based on the cDNA end-point PCR and sequencing data, the status of the cells with respect to rs1131341 was determined. All exon/intron boundaries with primers shown in Table 1 were analyzed, using the gDNA as a template. All primers, except primer NQO1 g6R (complementary to nucleotides in the 5’ part of the exon 6), were complementary to intronic/non-coding NQO1 DNA sequence. With these sets of analyses, we discovered a polymorphism in intron 1 (nt #248, according to NG_011504.2), present only in IMR-90 genomic DNA (Figure 7A), but not in the cancer cell lines (Figure 7B). To date, there is no data on the potential influence of this SNV on *NQO*1 splicing. In order to explore the potential influence of this polymorphism on splicing, novel primers, NQO1389460F and NQO1389460R, (Table 1) were constructed and analyses were performed as previously described. The existence of any alternative new splice variants which could be associated with this polymorphism, under the applied conditions could not be confirmed (Figure 7C).

#### 3.4.2. rs 689452, C+G, Is Present in Detroit 562 and Cal 27

**Figure 8:**
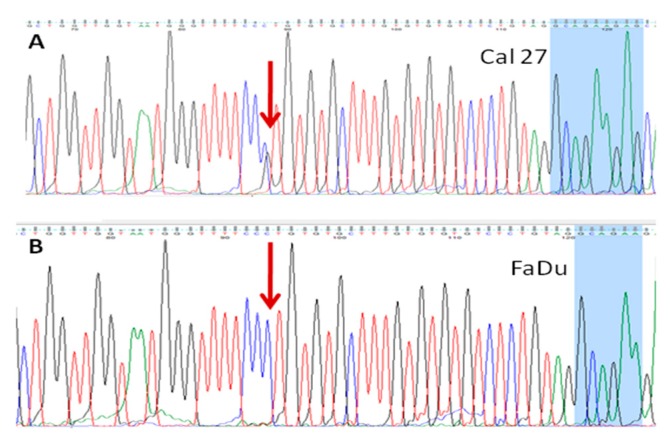
**A**. Single nucleotide variant, rs 689452, C+G in Cal 27 (also present in Detroit 562) is in intron 1, separated from the 5' part of exon 2 (labeled blue) by only 27 nts. **B**. In FaDu (shown by an arrow) and IMR-90 (not shown), the sequence was homozygous, C + C.

The presence of one more SNV in intron 1 (nt# 8070, according to NG_011504.2) was further discovered, in Detroit 562 and Cal 27 (Figure 8). The sequence variant (C+G) corresponds to rs 689452. In IMR-90 and FaDu, this position was homozygous, C+C, as shown on Figure 8. Based on the splice variants analyses, rs 689,452 does not influence the splicing of *NQO*1.

#### 3.4.3. Presence of rs1800566 in IMR-90 but Not in Cancer Cell Lines

**Figure 9:**
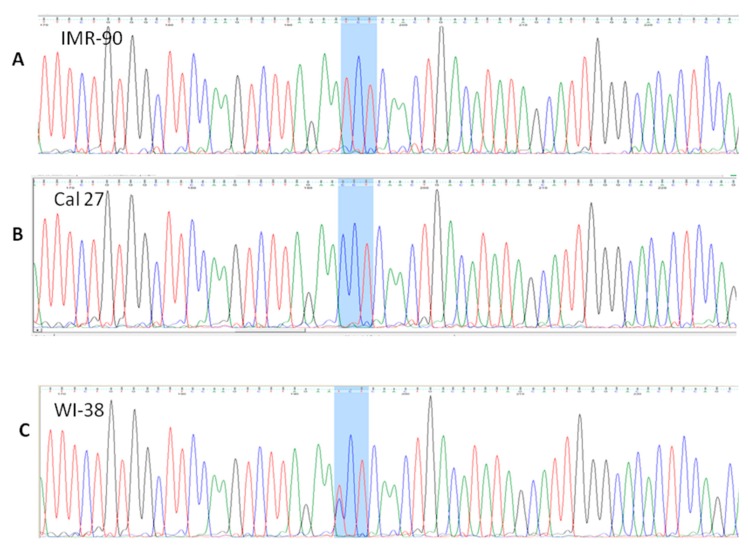
The presence of SNV rs1800566, in IMR-90 (NQO1*2/*2; (**A**)) and WI-38 (NQO1*1/*2; (**C**)). The homozygous triplet TCT, coding for Serine, replaces the CCT triplet coding for Proline, which was present in all cancer cell lines, as shown for Cal 27 (**B**).

The rs1800566 was detected only in IMR-90. This well-known polymorphism was present as a homozygous SNV–NQO1*2/*2 (nt #15389, according to NG_011504.2), leading to a change of the triplet CCT into TCT (Figure 9A). This is highly consequential, because this SNV missense variant, as described earlier, leads to replacing proline with serine at position 187. This polymorphism was shown as the one which influenced the structure of the NQO1 protein, making it highly unstable and catalytically compromised [43]. While it is known that IMR-90 expresses only traces of NQO1 protein, the data related to IMR-90 genotype, with respect to rs1800566, could not be found.

As human fibroblasts WI-38 express NQO1 but are not able to develop the NQO1-mediated redox cycle that could lead to HSP90 (Heat Shock Protein) inhibition upon β-Lapachone induction [44], it was presumed that these cells may have a genotype NQO1*1/*2. When we worked with WI-38 [45], the DNA was preserved from an early passage and kept frozen at −20 °C. Indeed, WI-38 are heterozygotes, NQO1*1/*2 (Figure 9C). This means that both commonly used cell lines are, regarding NQO1 activity, severely compromised and represent two totally different biological systems. All cancer cell lines were homozygous, P187P (Figure 9B – Cal 27) and the enzymatic activity of their NQO1 should be intact, at least with respect to rs1800566.

Finally, under the given conditions, we explored the NRF2/NQO1 axis on the protein level. In these analyses, TP53 was included. For the reasons explained earlier, western blots on nuclear and cytoplasmic fractions were performed.

### 3.5. Western Blot Analyses

#### 3.5.1. Analyses of NQO1

**Figure 10:**
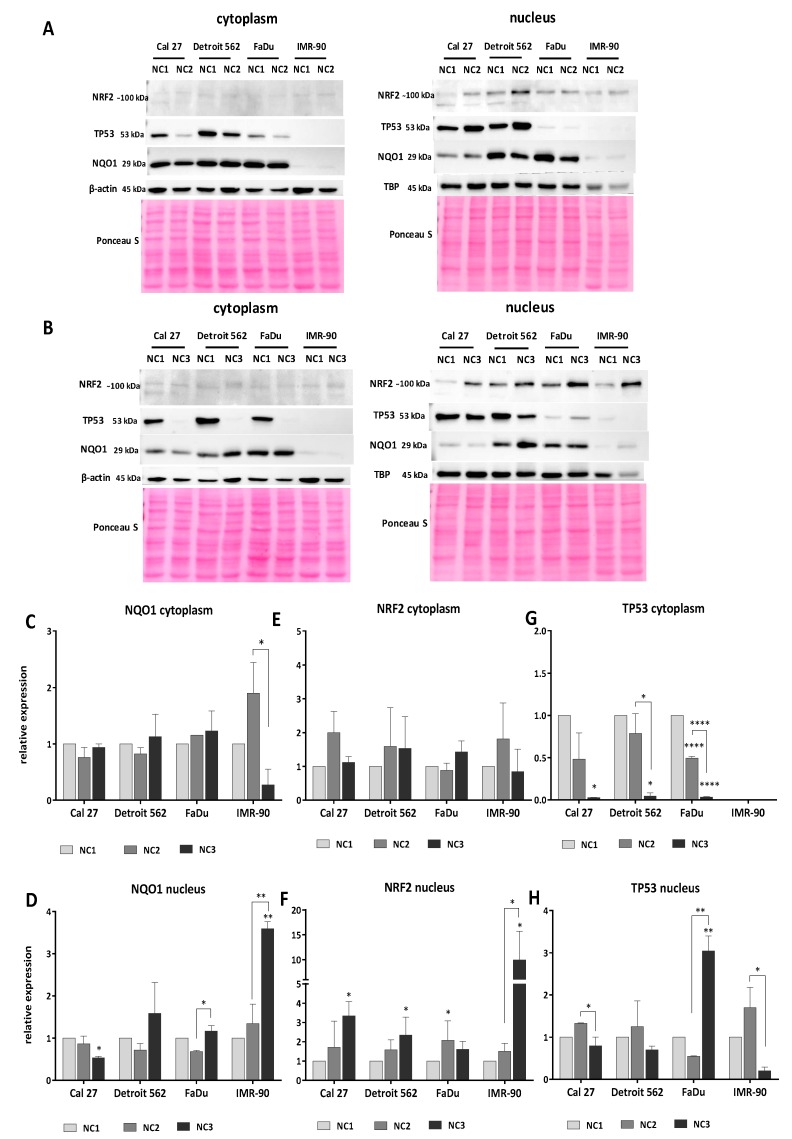
The expression of NQO1, NRF2 and TP53 in cancer cell lines and IMR-90. The representative western blots of cytoplasmic and nuclear NQO1, NRF2 and TP53 content under different nutritional conditions: (**A**) NC2 (low glucose + l-glutamine); (**B**) NC3 (no glucose and no l-glutamine). The relative expression is calculated as compared to the control condition (NC1 – high glucose + l-glutamine) for: **C**-cytoplasmic and **D**-nuclear NQO1; **E**-cytoplasmic and **F**-nuclear NRF2; **G**-cytoplasmic and **H**-nuclear TP53. One-way ANOVA with Tukey post hoc test was used to test the differences in relative expression of selected proteins under different nutrient conditions. The values are shown as the mean ± SD. n = 3. * *p* < 0.05; ** *p* < 0.01; *** *p* < 0.001; **** *p* < 0.0001.

Under NC1, the NQO1 signal was least visible in IMR-90. Although all three cancer cell lines had a considerably high level of NQO1 in both cellular fractions, a lower basal amount of NQO1 in Cal 27 nuclear fraction, as compared with FaDu and Detroit 562, was obvious on all our blots (Figure 10A,B). The level of NQO1 in the cellular fractions did not significantly change under the NC2 condition. However, some trends can be observed. There was a slight decrease of NQO1 in both cellular fractions of Cal 27 and Detroit 562 and an increase in both cellular IMR-90 fractions. The signal in FaDu was slightly increased in the cytoplasm, and, at the same time, there was a mild decrease in the nucleus (Figure 10C,D). Although under NC2 densitometry registers an obvious increase of NQO1 in both IMR-90 fractions, the intensity of the NQO1 signal remained very weak and far below the signal obtained in all cancer cell lines and under all tested conditions.

In all cancer cell lines, the more harsh condition of NC3 did not significantly affect the NQO1 cytoplasmic levels which remained close to the NQO1 control levels (NC1). In IMR-90, cytoplasmic level of NQO1 decreased (Figure 10C). Although not statistically significant as compared with the control (NC1), this decrease was quite intense when compared with cytoplasmic NQO1 expressed with respect to NC2 (*p* = 0.0107). IMR-90 responded with increased nuclear accumulation of NQO1 when exposed to NC3 (*p* = 0.0056). Under the same conditions, Cal 27 showed a nuclear decrease of NQO1 (*p* = 0.0457). Although the NC3 condition did not significantly affect the nuclear level of NQO1 in FaDu when compared with the control (NC1), a significant increase was recorded when compared to NC2 (*p* = 0.0178). An increased nuclear NQO1 level in NC3, when compared to NC2, was also observed in IMR-90 (*p* = 0.0085) (Figure 10D).

#### 3.5.2. Analyses of NRF2

The cytoplasmic expression of NRF2 in all four cell lines was weak and unaffected by nutritional conditions (Figure 10E). The nuclear NRF2 expression showed some differences. Although a nuclear increase of NRF2 in all cell lines under NC2 was recorded, it was statistically significant only in FaDu (*p* = 0.0414). Under NC3, a significant increase of NRF2 was recorded in the nucleus of the remaining three cell lines (NC1 vs. NC3: Cal 27 *p* = 0.039, Detroit 562 *p* = 0.0306, IMR-90 *p* = 0.0103). However, under these extreme conditions, it was not present in FaDu. With regard to all other cell lines, there was a difference between the nuclear amount of NRF2 under NC2 and NC3 in IMR-90 (*p* = 0.0142), which was manifested as a significant increase (Figure 10F).

It was proposed that NQO1 stabilizes both the wild-type and mutant-type TP53. There was a mixed situation—wild-type NQO1 and mutant TP53 in the cancer cell lines and mutant-type NQO1 and wt TP53 in IMR-90.

#### 3.5.3. Analyses of TP53

As expected, the wild type TP53 was not detected in the IMR-90 cytoplasm (Figure 10A,B). There was a significantly high level of the expressed mutant types of TP53 in the cytoplasm of the cancer cells in NC1. Cytoplasmic TP53 decreased in all cell lines under both NC2 and NC3. However, under NC2, the decrease reached a statistically significant level only in FaDu (*p* < 0.0001) (Figure 10G). The nuclear level of TP53 in Cal 27 and Detroit 562 exposed to NC2 for 48 h, was arround or slightly above the control (NC1) values (Figure 10H). Under NC3, the strong TP53 signal in the cytoplasm of the cancer cell lines literally disappeared (Figure 10B,G). It was present in the nucleus and showed very interesting trends in relation to the conditions applied. In Cal 27, IMR-90 and Detroit 562, the NC3 related TP53 signal was lower than in NC2. The decrease was significant for Cal 27 and IMR-90; NC2 versus NC3; *p* = 0.0411 and *p* = 0.0258. In FaDu, the signal of nuclear TP53 strongly increased and reached statistical significance (NC2 versus NC3; *p* = 0.0025) (Figure 10H).

The images on Figure 10 were obtained with 10 micrograms of proteins, under the same conditions. For improving the visibility of the bands corresponding to TP53 and NQO1 in IMR-90, a separate blot was made with the same amount of protein loaded (10 µg). For detection of the signals, software-controlled prolonged time exposure was used (Supplementary Figure S2).

#### 3.5.4. The General Picture of the NRF2-NQO1 Axis and TP53 at the Protein Level, in the Experimental Model

Under NC2, the NRF2-NQO1 axis did not seem to be activated in the cancer cell lines. This was because: a) Only FaDu exhibited a significant increase of NRF2 in the nucleus, which was joined with decreased NQO1 and TP53 signals in the nucleus; and b) Cal 27 and Detroit 562 had no significant increase of NRF2 in their nuclear fraction, nor did they exhibit a significant change in NQO1 expression. However, they had a slight increase of TP53 in their nuclei.

Under NC3, the NRF2-NQO1 axis seemed to have moderate activity in Detroit 562. This was because: (a) There was a significant increase of nuclear NRF2, associated with a slight increase of NQO1 in both cellular fractions; (b) Cal 27 had the strongest increase of nuclear NRF2 among all the cancer cell lines, which was not associated with the corresponding increase of NQO1; and (c) FaDu seems to be unresponsive.

There are opposite trends with respect to the TP53 nuclear signal when comparing Cal 27 and Detroit 562 to FaDu, under NC2 and NC3. Under NC2, the TP53 nuclear signal increased in Cal 27 and Detroit 562 and decreased in FaDu. Under NC3, the TP53 nuclear signal increased in FaDu and decreased in Cal 27 and Detroit 562.

The axis NRF2-NQO1 was active in IMR-90, the cell line with a wt TP53. This was because: (a) a slight and strong accumulation of NRF2 was observed in the nucleus under NC2 and NC3, respectively (Figure 10A,B,F); and (b) and was joined by a recordable accumulation of the NQO1 (S187S) protein, however unstable it was shown to be [43].

**Figure 11:**
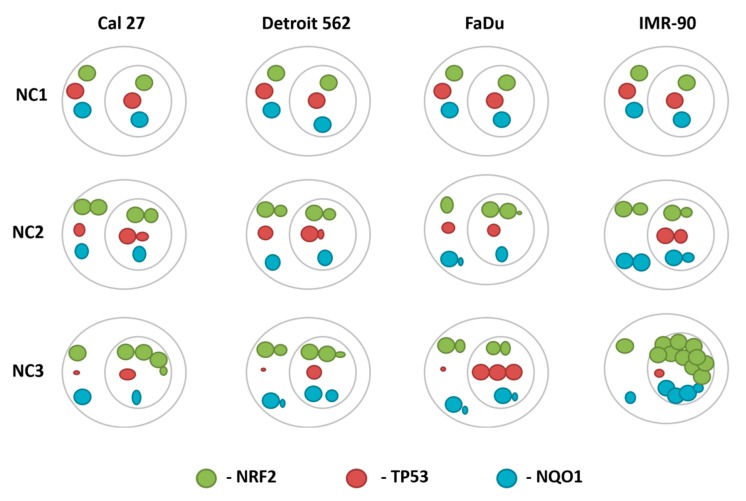
The cellular distribution and the rate of change of the NRF2, NQO1 and TP53 proteins in all four cell lines, under NC1-NC3.

The simplified graphic presentation of phenomena observed at the protein level is presented in Figure 11.

## 4. Discussion

The lack of nutrients leads to cellular oxidative responses. We wanted to explore the NRF2-NQO1 axis by inducing stress through incubating cancer cell lines in the following: A medium with a decreased concentration of glucose (1 g/L) (NC2); a medium with a complete lack of glucose (NC4) but supplemented with glutamine; and, in order to induce the strongest level of ROS, by creating an artificial situation, using a medium deprived of glutamine and glucose (NC3). However, due to trace amounts of glucose and glutamine in FBS, these cells were still exposed to a minimal concentration of glucose (~0.125 g/L ≈ 0.69 mM) [46] and glutamine (0.05 mM) [47].

The cellular viability, ROS generation and rate of cellular proliferation, were measured and compared under these four conditions, in three cancer cell lines, originating from HNSCC: FaDu, Detroit 562 and Cal 27. According to COSMIC and published papers, these three cell lines can be classified according to their TP53 mutation status [48]: 1. FaDu—heterozygous mutation leading to substitution of arginine with leucine at aa248 (R→L); 2. Cal 27—mutation (unknown zigosity status) leading to substitution of histidine with leucine at aa193 (H→L); 3. Detroit 562 mutation (unknown zigosity status) leading to the substitution of arginine with histidine at aa 175 (R→H). All these mutations are in the DNA-binding domain of the TP53 protein. Based on the cellular effects observed in the cancer cells, molecular-genetic experiments were chosen to be performed under conditions NC2 and NC3, to which fetal lung fibroblasts IMR-90 were also exposed. This cell line was used to show how MYC, after being transduced in IMR-90, makes IMR-90 addicted to glutamine [49]. Terashima et al. were able to stimulate NRF2 entering in the nucleus of these cells under the low glucose condition [50]. Thus, it was assumed that IMR-90 is likely to be sensitive to NC2 [49]. Many research groups were not able to show the expressed NQO1 protein in these cells in their native state [36]. However, there are also data showing the expressed, mature NQO1 in IMR-90 [51].

### 4.1. Cancer Cells and Fibroblasts IMR-90 Differ in Their Sensitivity to Nutrient Deprivation

We were aware that the chosen conditions were likely to reduce cellular viability and increase the level of ROS, but we could not predict to what extent that would happen. What was visible immediately was that the viability of the cancer cells under NC2 decreased significantly, but far less than under NC3. With respect to NC2 and the cancer cell lines, our results were in the strongest agreement with results published by Terashima et al., who presented a similar decrease of HepG2 viability cultured in a medium with low glucose (1 g/L) [50] In IMR-90 only, the NC2 condition had extremely strong effects with respect to the examined cellular parameters. In IMR-90 only, the effect of the NC2 condition was much stronger than in the cancer cell lines, confirming that IMR-90 are primarily dependent on glucose. The result obtained on IMR-90 is in agreement with data presented by Yunova et al., who also convincingly showed the sensitivity of IMR-90 to glucose deprivation [49]. With respect to glutamine, van den Heuvel et al. showed the insensitivity to glutamine depletion of normal human lung fibroblasts (NHLF)) cells originating from lung fibroblasts [52].

As shown on Figure 2A,D, among all four tested cell lines exposed to NC2, the viability of fibroblasts decreased below 50% (app. 80% in cancer cell lines). It did not decrease further when there was a lack of both glucose and glutamine (NC3). One possible explanation of this effect may be the high expression of glutamate/cystine antiporter solute carrier family 7 member 11 (SLC7A11, also called xCT) in IMR-90 cells [53]. In cancer cells, SLC7A11 mediates the efflux of intracellular glutamate, thereby rendering them metabolically less adaptable and more reliant on glucose for survival [54]. The drop in cellular viability of all cancer cell lines, contrary to IMR-90, was less pronounced under NC2 than under NC3. The proliferation rate of FaDu under NC4 was far below the proliferation rate in Cal 27 and Detroit 562. This indicates that there is a differential sensitivity to glutamine between FaDu and the other two cancer cell lines, when there is a lack of glucose.

Both FaDu and Cal 27 cells have been reported to express a high level of SLC7A11 [55]. The relative unresponsiveness of FaDu observed under NC4 may be explained, at least in part, by the observed high increase of nuclear NRF2 (*p* = 0.0414) (Figure 10F), joined with increased *NRF*2 transcription and the significantly increased generation of ROS (*p* < 0.0001) (Figure 2C), in response to NC2. Considering that NRF2 has been reported to induce *SLC7A11* expression in response to glucose starvation [51], it is conceivable that FaDu cells, in which exposure to NC2 upregulated NRF2, would under NC4 (complete lack of glucose, albeit supplemented with glutamine) have a more pronounced expression of *SLC7A*11 and efflux of glutamate that would prevent the recovery of cellular proliferation. Cal 27 cells, which were also reported to express a high level of *SLC7A*11 [55] but did not respond to NC2 by statistically significant upregulation of nuclear NRF2, significantly recovered cellular proliferation under NC4NC4), as contrasted with FaDu cells.

Under NC3, FaDu was the only cancer cell line which had a significant increase of NQO1 in the nucleus (NC2 vs. NC3 *p* = 0.0178). However, at the transcriptional level, significant changes relating to *NQO*1 transcriptional activity in FaDu were not detected, neither under NC2 (accurately measured), nor under NC3 (approximated).

Cal 27 was the cell line with the lowest basal level of NRF2 (Figure 10A,B, NC1—nuclear fraction). Probst et al. were able to show that the cell lines with high basal NRF2 activity exhibited little or no increase in *NQO1* mRNA levels following NRF2 activation with the compound RTA 405 [56]. We think this fact can explain part of our results obtained on NQO1 transcripts.

### 4.2. Importance of Defining the SNVs, in a Given Experimental Model

When *NQO*1 transcripts were quantified, a significant increase of the *NQO*1 transcript was shown only in IMR-90 and Cal 27 but not in FaDu (under NC2) and Detroit 562. Unfortunately, however high was the expression in IMR-90, there was a minimal amount of NQO1 protein. The level of the *NQO*1 transcript in IMR-90 was strong and it did not differ from the mRNA NQO1 signal in the cancer cell lines. A majority of research groups had not detected NQO1 protein in IMR-90 (at least not under restful conditions). However, it seems that nobody reported the data on the IMR-90 *NQO*1 genotype. After performing the DNA sequencing, it was concluded that this cell line is a NQO1*2/*2 homozygote (rs180566). Some inconclusive data obtained on another human fibroblast cell line, WI-38, encouraged us to analyze it in the same way. It was shown that WI-38 also bears rs180566 but is a heterozygote (NQO1*1/*2) cell line. Thus, at least with respect to NQO1, IMR-90 and WI-38 are not normal fibroblasts. Accordingly, any conclusion obtained on one cell line cannot be automatically translated to another (or any other) cell line. Regarding rs180566, detailed modeling performed by Lienhart et al. [43] did not provide a structure-based explanation for the lower enzymatic activity of NQO1 P187S. A plausible explanation given in an in vivo model by Tsvetkov et al., should be considered in light of discovering that E3 ligase STUB1/CHIP (C terminus of Hsc70-interacting protein) regulates the NQO1 protein level through ubiquitination and degradation [57]. The heterozygote P187S (rs180566) was shown to be a stronger STUB1 interactor with an increased susceptibility to ubiquitination by the E3 ligase STUB. Thus, we concluded that the homozygote, S187S, may have even stronger affinity for STUB1. This finally resulted in an almost undetectable NQO1 in IMR-90, notwithstanding the increase of *NQO*1 transcriptional activity (Figure 3; Figure 4) under NC3 which was associated with a physiological increase of nuclear NRF2 (Figure 10B). An increased sensitivity of IMR-90 to NC2 may be a consequence of lacking a strong mechanism for influencing the NAD(P)+/NAD(P)H redox balance during the stress-related events, due to catalytically insufficient NQO1.

### 4.3. FaDu Proliferative Potential with Respect to GLS1 and Decreased Sensitivity to Glutamine When Deprived of Glucose

It was very interesting to see that that the effect of glutamine on FaDu, with respect to proliferative potential when deprived of glucose, exerts a less positive effect than with Cal 27 and Detroit 562 (Figure 2B). The enzyme, Glutaminase 1 (GLS1), enables malignant cells to undergo increased glutaminolysis and utilization of glutamine as an alternative nutrient. A recently published study has clearly shown that the expression of *GLS*1 in FaDu is far less prominent than in Detroit 562 cells [58]. Thus, the capacity of FaDu for glutamine utilization seems to be constitutively decreased. Although we did not measure *GLS*1, we hypothesized that the less prominent rescuing effect of glutamine on FaDu than on Detroit 562 and Cal 27, in the absence of glucose, occurs as a result of low basal expression of *GLS*1 In IMR-90, GLS1 is also present at a low level and its pharmacological inhibition does not change the level of intracellular ATP [59]. Sandulache et al. discovered that a majority of HNSCC cancer cell lines show a dependence on glucose and not glutamine [60]. This study also referred to FaDu. However, FaDu, as contrasted with all of the other 14 tested HNSCC (Detroit 562 and Cal 27 were not included in the panel), did not exert a similar rate of sensitivity to non-metabolizable D-glucose analogues. The data was: IC_50_ 10.90 (FaDu) versus IC_50_ 0.79 with UMSCC22B—the most sensitive cell line.

### 4.4. NQO1 and Its Splice Variants—TaqMan Probes Validation

Regardless of the conditions applied, three *NQO*1 transcript variants were detected. Based on WB analyses, only the longest one, TV1, as compared with the other two, TV3 and TV4, seemed to translate in the mature protein. The change of the ratio TV1/TV3 was measured. We have shown that only in Cal 27, under NC3, did this ratio increase. It decreased in the other three cell lines. It is unfortunate that the *NQO*1 transcript fold change under NC3 in FaDu could not be accurately measured (Figure 3F), where there was a significant decrease of the TV1/TV3 ratio (Figure 6). Based on the Ct values, we estimated that the *NQO*1 transcription, under NC3, does not significantly vary from the control condition (NC1) (Figure 3). The change of TV1/TV3 ratio was not influenced by rs1131341 [31], as all cell lines had the same genotype with respect to this SNV. Thus, we concluded that the change TV1/TV3 depends on both conditions applied and the cell-type specific response, which clearly differentiated Cal 27 from other cell lines [61]. It is possible that, through this mechanism, this cell line compensates NQO1 basal activity, which is significantly lower (90 U) than the NQO1 activity in Detroit 562 (790 U) and FaDu (1400 U) [36]. It also raises a question with respect to compensatory mechanisms associated not only with the level of transcription, but also with splicing, when there is a high ROS generation. Cal 27, the cell line with the lowest NQO1 activity under basal conditions, had the strongest increase of NQO1 transcript and adjusted the TV1/TV3 ratio in favor of TV1. FaDu, the cell line with the highest NQO1 activity under basal conditions, had no increase of NQO1 transcript and reduced the TV1/TV3 ratio.

### 4.5. Activation of NRF2 and Sensitivity to Glutamine

In the Results section, we presented how some cellular parameters are indicative of a different sensitivity to glutamine, which is higher in Cal 27 and Detroit 562, and lower in FaDu. How is that to be explained, in addition to the previously given explanation relating to the cellular transporters? First, it should be noted that the intensity of the nuclear and cytoplasmic NQO1 signal was far less prominent in Cal 27 under NC1, than in the other two cancer cell lines (Figure 10A,B). The same phenomenon was shown in Li’s paper [36]. The reason for less NQO1 protein is not the presence of rs18066, as Cal 27 are NQO1*1/*1. One possibility may be that these cells have a genuinely less active NRF2-KEAP1 pathway. Indeed, Romero et al. have suggested NQO1 as a suitable biomarker for NRF2 activation, when researching a human KRAS-mutant lung adenocarcinoma (LUAD) [62]. In our experimental model, this was not shown. However, one can speculate that the NRF2-KEAP1 axis, weaker in Cal 27 than in FaDu and Detroit 562, can be strongly activated. Based on the results presented, Cal 27 cells indeed have a stronger potential for activating the NRF2-NQO1 axis than the other two cancer cell lines. The activation of the NRF2-KEAP1 axis, which was shown to be, according to the majority of the parameters measured, highly dependent on glutamine [63], needs to be explored further in dynamic and not end-point experiments.

### 4.6. TP53 and Its Potential to Influence Phenomena Observed

Regarding the influence of MT TP53 on NRF2, there are many data, but there are no conclusions. Lisek et al. showed that mutant TP53 increases NRF2 localization to the nucleus of cancer cells, where it redirects NRF2 to ARE elements of specific genes to activate their transcription. Conversely, it sequesters NRF2 from other targets, leading to their downregulation [64]. Kalo et al. have shown that induction of stress in HCT116 cells bearing TP53 mutant R273H results in NRF2 nuclear accumulation. However, the transcription of target genes was induced to a much lesser extent than in HCT116 without TP53 activity (TP53−/−). They also showed that the down-regulation of endogenous mutant TP53, results in increased mRNA levels of NQO1 and Hem Oxygenase (HMOX-1). Thus, they proposed that MT TP53 promotes the survival of cells with high level ROS [65]. Under NC3, a decrease of nuclear TP53 in Cal 27 is related to an increased transcription rate of *NQO*1. At the same time, the strong increase of TP53 in the nuclei of FaDu under NC3 may be associated with its silencing effect on *NQO*1 transcription (as estimated). This would be in accord with Kalo’s results, at least at the level of transcriptional activity of only *NQO*1. When we measured the transcriptional activity of *HMOX*-1, a tremendous transcriptional activity of *HMOX*-1 in IMR-90 and FaDu under NC3 was recorded (data not shown). This does not necessarily mean that this increased transcription relies on NRF2, as we need to perform chromatin immunoprecipitations and selective silencing in order to understand the molecular mechanisms involved.

One important aspect of the events which seems connected to accumulating phenomena related to nutritional stress and TP53, may be on an entirely different level. The ubiquitin ligase Mdm-2, which mediates TP53 degradation in the proteasome [66], is a transcriptional target of TP53 [67]. Qie et al. [68] reported the upregulated transcription of MDM2 in Hep3B cells cultured in a glutamine free medium, despite the homozygous deletion of TP53, pointing to the existence of alternative regulatory mechanisms. Considering the role of MDM2 in TP53 degradation, if the reported increase in MDM2 transcripts translates to an increased protein level, one would expect a decrease in the TP53 upon glutamine deprivation in TP53 expressing cells. Although it is seemingly surprising, a redistribution of TP53 among cellular compartments (cytoplasm and nucleus) was observed, rather than its decrease in the cells deprived of glutamine (NC3; Figure 10B). A possible explanation for this effect may be the nuclear retention of TP53 due to its poly(ADP-ribosyl)ation that prevents TP53 interaction with the nuclear export receptor CRM1 [69]. Chiodi et al. recently reported that glucose and/or glutamine deprivation causes very rapid PARP-1 activation and protein poly(ADP-ribosyl)ation [70]. This is consistent with the intracellular distribution of TP53 that was observed in all the tested cells grown in a low glucose medium (NC2) or glucose- and glutamine-free medium (NC3) (Figure 10C). Namely, in all cell lines, TP53 was less abundant in the cytoplasm of the cells grown under NC2 and almost completely absent in the cytoplasm of the cells simultaneously deprived of glucose and glutamine (NC3). Contrary to cytoplasm, a significant amount of TP53 was present in the nuclear fraction. The retention of TP53 in the nuclei of all cells exposed to NC2 and NC3, regardless of their TP53 mutational status, is consistent with the report that three amino acids (E258, D259 and E271) are the targets of poly(ADP-ribosyl)ation, and that TP53 failed to get poly(ADP-ribosyl)ated only when all three of them were replaced with alanine [69]. Therefore, poly(ADP-ribosyl)ation can retain both mutant and WT TP53 in the nucleus.

The importance of NQO1 activity for TP53 accumulation in the cell strongly argues in favor of the involvement of poly(ADP-ribosy)lation in TP53 stabilization. This is because it was shown that the inhibition of NQO1 activity by dicumarol induces proteasomal degradation of WT and MT p53 [18]. The enzymatic activity of NQO1 is needed for generating NAD^+^, which is a co-substrate for PARP-1 that transfers ADP-ribose moieties from NAD^+^ to proteins including TP53 [71]. Experimentally induced PARP-1 or NAD^+^ deficiency has been reported to result in a significantly reduced level and activity of TP53 [72]. Therefore, the accumulation of TP53 in the nuclei of the cells exposed to glucose and glucose/glutamine deprivation (NC2 and NC3), may be partly mediated by NQO1 (and other oxidoreductases like WOX1 [73]. Asher et al. [18] suggested that, considering the presence of several putative TP53-binding elements in *NQO*1 promoter, *NQO*1 may belong to TP53-inducible genes involved in a positive autoregulatory loop that regulates the level of TP53. Therefore, the mutational status of TP53 may have a profound influence on NQO1 expression in cells that are exposed to nutritional stress.

## 5. Conclusions

After modulating FaDu, Cal 27 and Detroit 562 for the vital cellular parameters of viability, proliferation and generation of ROS while cultivating them under four different nutritional conditions (NC1-NC4), some general conclusions can be drawn: (a) In relation to all three parameters analyzed, these cell lines showed sensitivity to glucose deprivation; (b) when having available minimal amounts of glucose and glutamine (NC2) FaDu, Cal 27, Detroit 562 responded strongly with respect to all three parameters; (c) only FaDu cells showed an increased need for glucose, not glutamine (NC3 versus NC4), for sustaining replication activity. A strong increase of ROS influences the NRF2-NQO1 axis in these cells in a fashion which is apparently cell-type specific. When considering the activation of the axis through NRF2 nuclear accumulation, the strongest response under a milder condition (NC2) was recorded in FaDu, associated with a decrease of the nuclear TP53 signal. Under harsh conditions (NC3), Cal 27 and Detroit 562 responded with NRF2 nuclear accumulation, associated again, with a decrease of the nuclear TP53 signal. Obtaining the same phenomena under different conditions (Cal 27 and Detroit 562 versus FaDu) pointed out the differences in response to identical stress, which correlates with the fact that only FaDu cells did not recover their replicative potential when deprived of glucose, in the presence of glutamine.

When considering the activation of the axis through an increase in *NQO*1 transcription, only Cal 27 responded adequately, through an increase in the *NQO*1 transcription rate and a modulation of alternative splicing, in favor of TV1. FaDu responded in an entirely different fashion, with a decrease in *NQO*1 transcript and a modulation of alternative splicing, in favor of TV3. These responses may be consequential with respect to NQO1 enzymatic activity in Cal 27 and FaDu, which was previously shown to be 15 times higher in FaDu. Thus, the whole response of the NRF2-NQO1 axis to stress should be considered in the broader context of the cellular background.

Detroit 562 is the cell line which moderately activated its NRF2-NQO1 axis, on both the transcriptional and protein level. It is the only cancer cell line which had no significant increase of ROS, under NC2.

Fibroblasts IMR-90 were entirely dependent on glucose. These cells exhibited a physiological cellular response relating to the activation of NRF2-NQO1 axis during nutritional stress, which resulted with hardly detectable NQO1 signals when compared to the cancer cell lines. IMR-90 are homozygous – NQO1*2/*2, with respect to rs1800566.

Without making the genotyping in respect to rs1800566, we would still be confident that IMR-90 indeed are *NQO*1 non-expressing cells. According to all available data, their rs1800566 genotype directs an extremely high rate of the NQO1 protein degradation, although the NRF2-NQO1 axis in these cells activates during nutritional stress.

Thus, when making important conclusions on the strength of the NRF2-NQO1 axis through NQO1 protein level/enzymatic activity, the status of rs1800566, as well as specifics of the cellular background, are always relevant and must be considered.

## Figures and Tables

**Table 1:** The positions and composition of primers used for analyses of *NQO*1.

Primers	Primer Sequences	Ref. Seq.	Primer Position	Amplicon Size
	GAPDH primers			
GAPDH1	5′AACGGATTTGGTCGTATTGGGC3′	NM_002046.7	101–122	600 bps
GAPDH2	5′AGGGATGATGTTCTGGAGAGCC3′		679–700	
GAPDH2	5′AGGGATGATGTTCTGGAGAGCC3′	NG_007073.2	3145–3166	644 bps
GAPDH3	5′AAGCTGACTCAGCCCGCAAAGG3′		2523–2545	
	**NQO1 primers**			
	**cDNA**			
NQO1 F	5′GTCGGACCTCTATGCCATGA3′	NM_000903.3	238–257	TV1—685 bps
				TV2—583 bps
				TV3—571 bps
NQO1 R	5′GTCAGTTGGGATGGACTTGC3′		905–922	TV4—469 bps
NQO1389460 F	5′CAGCTCACCGAGAGCCTAGT3′		3756	
NQO1389460 R	5′CATGGCATAGAGGTCCGACT3′		237–257	221 bps
	**Genomic DNA**			
NQO1 g1F	5′CACACACACCCCTACAATCCCC3′		(−246)–(−225)	509 bps
NQO1 g1R	5′CCAGGTCCCTAATCTCTTCCC3′		243263	
NQO1 g2F	5′ACATTTCTGGCTACAGGAGATGGA3′		78827905	704 bps
NQO1 g3	5′GTCAGTTGGGATGGACTTGC3′		8573–8594	
NQO1 g4F	5′CAGCTCACCGAGAGCCTAGT3′		11299–11318	361 bps
NQO1 g4R	5′GAAATCCATGTAATACTGCACCT3′	NG_011504.2	11641–11659	
NQO1 g5F	5′AGTTGGCTGACCAAGGACAA3′		13285–13304	591 bps
NQO1 g5R	5′CCCTGCATCAGGACAGACC3′		13855–13875	
NQO1 g6F	5′TAGCTCAGGGGAGCCAAAGT3′		15104–15124	693 bps
NQO1 g6R	5′TGAATTCCCCTGAAGGTTCGT3′		1577715796	
NQO1 g1F	5′TGGTAACGGCTAGGTAGAGGG3′		(−246)–(−225)	509 bps
NQO1 g1R	5′AGCCCAGTCGGATTTTGGTT3′		243–263	

## Supplementary Materials

The following are available online at https://www.mdpi.com/2073-4409/8/9/1001/s1, Figure S1: Representative image of *NQO*1 transcript variants after 28 PCR cycles. Figure S2. TP53 and NQO1 in IMR-90.

## Abbreviations

AMPK	5′AMP-Activated Protein Kinase
ARE	Antioxidant Response Element
ATCC	American Type Culture Collection
ATP	Adenosine Triphosphate
BrdU	5-Bromo-2′-Deoxyuridine
COSMIC	Catalogue of Somatic Mutations In Cancer
DCFH-DA	2ʹ,7ʹ-Dichlorofluorescin Diacetate
DMEM	Dulbecco’s Modified Eagle’s Medium
EDTA	Ethylenediaminetetraacetic Acid
ER	Endoplasmic Reticulum
EtdBr	Ethidium Bromide
FAD	Flavin Adenine Dinucleotide
FBS	Fetal Bovine Serum
G6PD	Glucose-6-Phosphate Dehydrogenase
GAPDH	Glyceraldehyde-3-Phosphate Dehydrogenase
gDNA	Genomic DNA
GLS1	Glutaminase 1
HCT116	Human Colorectal Carcinoma
HIF-1α	Hypoxia-Inducible Factor, Alpha Subunit
HMOX-1	Heme Oxygenase 1
HNSCC	Head and Neck Squamous Cell Carcinoma
HPRT1	Hypoxanthine Phosphoribosyltransferase 1
HSP90	Heat Shock Protein 90
IR-C	Intron Retention Complex
IR-S	Intron Retention Simple
KEAP-1	Kelch-Like ECH-Associated Protein 1
LUAD	Lung Adenocarcinoma
Mdm-2	Mouse Double Minute 2 Homolog
MT	Mutated
NADH/NAD+	Nicotinamide Adenine Dinucleotide
NADPH/NADP+	Nicotinamide Adenine Dinucleotide Phosphate
NC	Nutritional Condition
NC1	Nutritional Condition 1
NC2	Nutritional Condition 2
NC3	Nutritional Condition 3
NC4	Nutritional Condition 4
NFE2L2 (NRF2)	Nuclear Factor (Erythroid-Derived 2)-Like 2
NHLF	Normal Human Lung Fibroblasts
NQO1	NAD(P)H:Quinone Oxidoreductase 1
PARP-1	Poly (ADP-Ribose) Polymerase 1
PCR	Polymerase Chain Reaction
PPP	Pentose Phosphate Pathway
ROS	Reactive Oxygene Species
RT-qPCR	Reverse Transcription-QuantitativePolymerase Chain Reaction
SIRT1	Sirtuin 1
SLC7A11	Solute-Like Carrier Family 7, Member 11
SLC7A3	Solute-Like Carrier Family 7, Member 3
SNP	Single-Nucleotide Polymorphism
SNV	Single Nucleotide Variant
TBP	TATA-Box Binding Protein
TBS	Tris-Buffered Saline
TBST	Tris Buffered Saline with Tween-20
TE buffer	Tris-EDTA Buffer
TP53	Tumor Protein P53
TV	Transcript Variant
WOX1	WUSCHEL Related Homeobox 1
WT	Wild-Type
