# Supplementary figures and images for: Nutritional Stress in Head and Neck Cancer Originating Cell Lines: The Sensitivity of the NRF2-NQO1 Axis
Source: Cells. 2019 Aug 29;8(9):1001. doi: 10.3390/cells8091001 (PMC6769674; doi:10.3390/cells8091001)

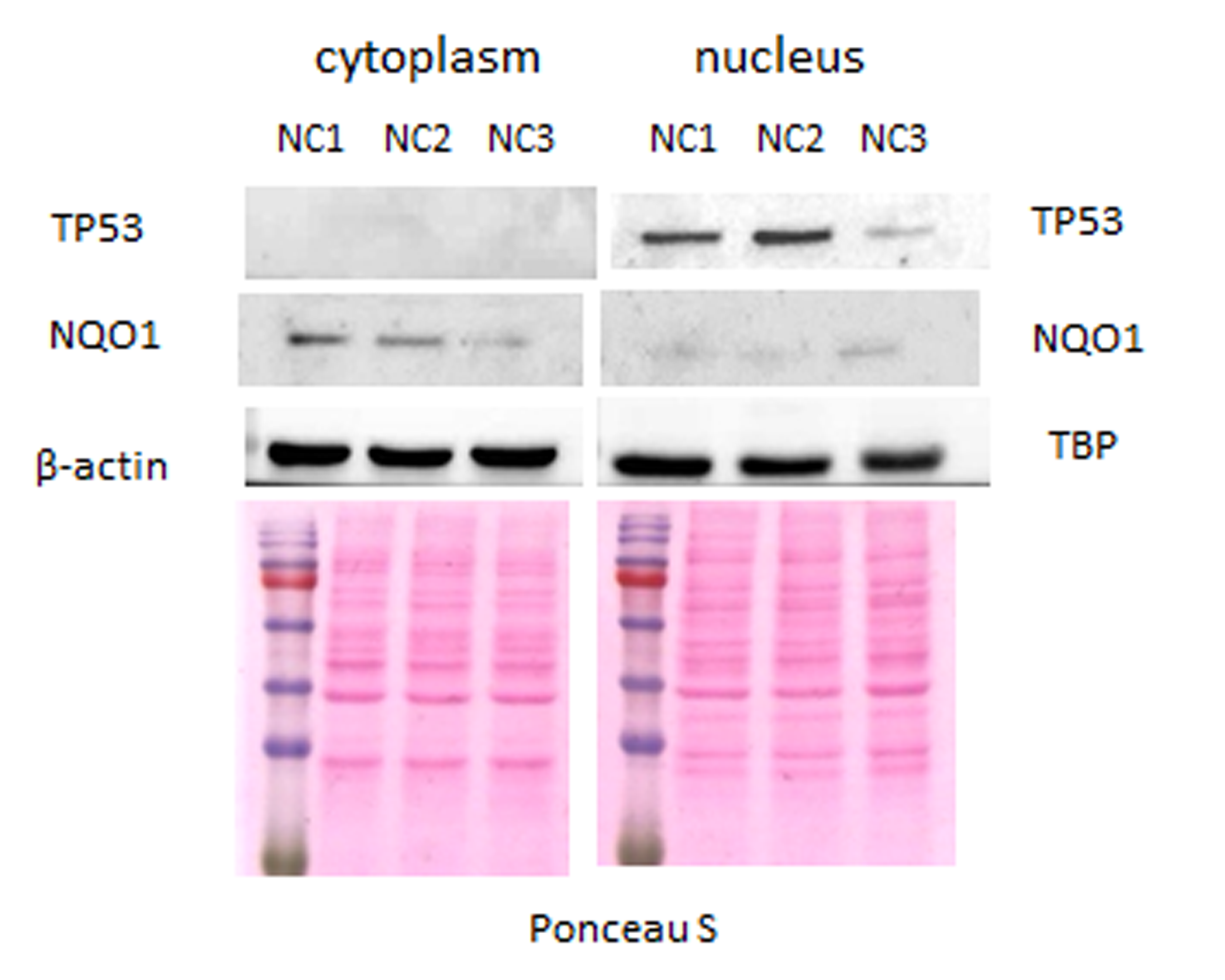

Supplement: Supplementary file 1 [file cells-08-01001-s001.zip › Supplementary Fig 2.tif]

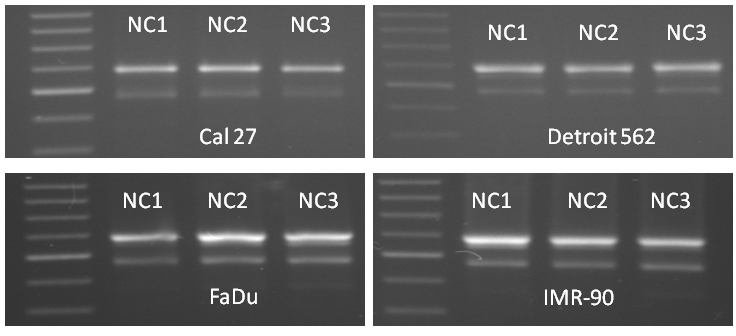

Supplement: Supplementary file 1 [file cells-08-01001-s001.zip › Supplementary Fig 1.tif]
